# Protective role of ABCA1 in ischemic preconditioning is mediated by downregulation of miR-33-5p and miR-135-5p

**DOI:** 10.1038/s41598-021-91982-x

**Published:** 2021-06-15

**Authors:** Hye Youn Sung, Eun Nam Choi, Jihye Han, Yun Ju Chae, Sun-Wha Im, Hee-Sun Kim, Eun-Mi Park, Jung-Hyuck Ahn

**Affiliations:** 1 Department of Biochemistry, College of Medicine, Ewha Womans University, 25 Magokdong-ro 2-gil, Gangseo-gu, Seoul, 07804 Korea; 2 Genomic Medicine Institute, Medical Research Center, Seoul National University, Seoul, Korea; 3 Department of Molecular Medicine, College of Medicine, Ewha Womans University, Seoul, Korea; 4 Department of Pharmacology, College of Medicine, Ewha Womans University, 25 Magokdong-ro 2-gil, Gangseo-gu, Seoul, 07804 Korea

**Keywords:** Neuroscience, Molecular medicine, Neurology

## Abstract

Ischemic preconditioning (IPC) significantly reduces ischemia–reperfusion injury in the brain by inducing ischemic tolerance. Although emerging evidence suggests that microRNAs (miRNAs) contribute to the pathogenesis of brain ischemia and IPC-induced neuroprotection, the role of miRNAs and their underlying mechanisms are still unclear. IPC was induced in male C57BL/6 mice by brief bilateral common carotid artery occlusion. After 24 h, mice underwent transient middle cerebral artery occlusion followed by 3 h of reperfusion. Expression levels of messenger RNAs (mRNAs) and proteins were examined in the ipsilateral cortex, and mimics and inhibitors of selective miRNAs were transfected into Neuro-2a cells before oxygen–glucose deprivation (OGD). Post-IPC miRNA expression profiling identified neuroprotection-associated changes in miRNA expression in the ipsilateral cortex after ischemic stroke. Among them, miR-33-5p and miR-135b-5p were significantly downregulated by IPC. Inhibition of miR-33-5p and miR-135b-5p expression protected Neuro-2a cells from OGD-induced apoptosis. Inhibition of these two miRNAs significantly increased mRNA and protein levels of ATP-binding cassette subfamily A member 1 (ABCA1), and a binding assay showed that these two miRNAs showed specificity for *Abca1* mRNA. Overexpression of ABCA1 decreased the *Bax/Bcl2* mRNA ratio and activation of caspase-9 and caspase-3, whereas knockdown of ABCA1 expression increased the *Bax/Bcl2* mRNA ratio and the percentage of Neuro-2a cells with a loss of mitochondrial membrane potential after OGD-treatment. In conclusion, ABCA1 expression is regulated by miR-33-5p and miR-135b-5p. Increased ABCA1 expression following IPC exerts a protective influence against cerebral ischemia via suppression of a mitochondria-dependent apoptosis pathway.

## Introduction

Cerebral ischemia is a leading cause of death and disability worldwide, and it occurs as a result of an interruption in the brain's blood supply of oxygen and nutrients and results in brain tissue damage and loss of function.

Ischemic preconditioning (IPC) refers to a process whereby an endogenous neuroprotective brain response is induced using sublethal ischemic exposure prior to an otherwise lethal ischemic insult, allowing the brain to better tolerate the otherwise severe damage^1^. Although IPC initiates a cascade of intercellular signaling pathways related to neurovascular protection, anti-inflammatory action, reduced excitotoxicity, and metabolic protection^2^, the precise molecular mechanisms responsible for this neuroprotection remain uncertain.

MicroRNAs (miRNAs) are small non-coding RNA molecules of ~ 22 nucleotides that regulate gene expression at the post-transcriptional level via destabilization and translational inhibition of their target messenger RNAs (mRNAs)^3^. Furthermore, miRNAs are abundant in the nervous system where they serve prominent roles in development, neural plasticity, and in neural disorders as well^4^. Emerging evidence shows that miRNAs contribute to the pathogenesis of brain ischemia and the neuroprotection induced by IPC by acting as both negative and positive regulators of cell survival^5,6^. Recent studies have documented changes in miRNA expression profiles in response to ischemia using both experimental cerebral ischemia and/or IPC models, suggesting that miRNA-mediated post-transcriptional inhibition may be important for regulating gene expression under ischemic conditions^6–8^. Experimental manipulation of specific miRNAs has defined their targets and the mechanisms underlying their effects for the purpose of identifying promising new treatment targets for stroke using different models of IPC^7,9,10^. However, due to different experimental designs, different IPC stimuli, and diverse in vivo and in vitro models, the complexity of IPC-induced neuroprotection through miRNAs and their targets makes it difficult to understand the extent of their contributions.

In this study, we investigated the expression patterns of miRNAs after IPC in a mouse cerebral ischemia model. This expression analysis identified three miRNAs, miR-33-5p, miR-135b-5p, and miR-551b-3p, that were selectively downregulated after IPC. Suppressing the expression of these miRNAs protected neuronal cells from ischemia-induced apoptosis. Two of these miRNAs, miR-33-5p and miR-135b-5p, targeted ATP-binding cassette subfamily A member 1 (ABCA1), and enhanced ABCA1 expression through their downregulation in Neuro-2a cells, and protected the cells from ischemia-induced apoptosis. Here, we suggest that targeting miR-33-5p and miR-135b-5p may be a potential treatment strategy for preventing neuronal damage after ischemic stroke.

## Materials and methods

### Animals

Experiments were performed in male C57BL/6 mice (age 10 to 11 weeks weighing 24–26 g, Orient Bio Inc., Seongnam, Republic of Korea). For the measurements of mRNA levels, miRNAs, and protein levels (n = 6 per group) in the brain, total 30 mice were used and included in the data analysis. The number of animals used for the study was determined based on the previous study^11^ and minimized to reduce animal suffering. All procedures were approved by the Institutional Animal Care and Use Committee at the Medical School of Ewha Womans University (11-0170) and conformed to international guidelines on the ethical use of animals. The number of animals used for the study was minimized to reduce animal suffering. The study was carried out in compliance with the ARRIVE guidelines.

### IPC by bilateral common carotid artery occlusion (BCCAO)

Procedures for BCCAO preconditioning have been established and published previously^11^. Briefly, mice were anesthetized with isoflurane (1.6–2.0%) and a fiber optic probe was glued to the right parietal bone (2 mm posterior and 5 mm lateral to bregma) and connected to a laser-Doppler flowmeter (Periflux System 5010, Perimed, Sweden). Cerebral blood flow (CBF) was continuously recorded using a computer-based data acquisition system (Perisoft, New South Wales, Australia). IPC was induced by 3 episodes of 1 min occlusion of both common carotid arteries, each followed by 5 min of reperfusion. Wounds were closed and animals were returned to their cages. In the sham-operated mice, the carotid arteries were exposed for 15 min without occlusion (sham). Control animals did not receive any surgical operation (control). Animals were assigned to groups randomly by computerized random number generators.

### Transient middle cerebral artery occlusion (MCAO)

Time window of BCCAO IPC paradigm for ischemic stroke was previously defined^12^, therefore, we confirmed the tolerance against ischemic stroke at 24 h after BCCAO IPC in this study. Mice were reanesthetized with isoflurane 24 h after BCCAO (IPC + MCAO group) or sham operation (sham + MCAO group), which were blinded, and the fiber optic probe was reattached to the right parietal bone (2 mm posterior and 5 mm lateral to bregma) and connected to a laser-Doppler flowmeter. CBF was continuously recorded during MCAO and reperfusion periods.

Techniques for transient MCAO with an intravascular suture have been described previously^12,13^. Briefly, a 6–0 silicon-coated black monofilament surgical suture (Doccol Cooperation, Redlands, CA, USA) was inserted into the exposed right external carotid artery, advanced into the internal carotid artery, and wedged into the circle of Willis to obstruct the origin of the middle cerebral artery (MCA). The filament was left in place for 30 min and then withdrawn to re-establish CBF. Only animals that exhibited a reduction in CBF > 85% during MCAO and in which CBF recovered by > 80% after 10 min of reperfusion were included in the study. Rectal temperature was maintained at 37.0 ± 0.5 °C using a thermostatically controlled heating pad, both during surgery and during the recovery period until consciousness was regained. After 3 h reperfusion, mice were euthanized, and the ipsilateral cortex of brain was isolated for the experiments because the protective effect of IPC was limited in the cortex^11^.

### Cell culture

Mouse neuroblastoma cell line Neuro-2a was purchased from American Type Culture Collection (ATCC no. CCL-131; Manassas, VA, USA) and cultured in Dulbecco's modified Eagle's medium (Thermo Fisher Scientific, Waltham, MA, USA) supplemented with 10% fetal bovine serum (FBS, Thermo Fisher Scientific), 100 U/ml penicillin (Thermo Fisher Scientific), and 100 μg/ml streptomycin (Thermo Fisher Scientific) in an atmosphere of 95% humidified air and 5% CO_2_ at 37 °C.

To mimic in vitro ischemic-like condition, Neuro-2a cells were exposed to oxygen–glucose deprivation (OGD). The cells were washed with phosphate-buffered saline (137 mM NaCl, 2.7 mM KCl, 10 mM Na_2_HPO_4_, 1.8 mM KH_2_PO_4_, pH 7.4) and replaced with glucose-free RKRB buffer (115 mM NaCl, 1 mM KH_2_PO_4_, 4 mM KCl, 1 mM MgSO_4_, 1.25 mM CaCl_2_, 25 mM NaHCO_3_, pH 7.4). The cells were then placed in hypoxia chamber (Astec, Fukuoka, Japan) for 5 h at 5% CO_2_, 1% O_2_ and 94% N_2_. After treatment, Neuro-2a cells were returned normoxic condition with normal culture media for 1 h for reperfusion. Control cells were not exposed to hypoxic stimulation.

In order for IPC in vitro, Neuro-2a cells overnight post-seeding were placed in a hypoxia chamber (5% CO_2_, 1% O_2_ and 94% N_2_, 37 °C) for 1 h prior to a main hypoxic insult. After 1 h equilibration, the cells underwent 5 h hypoxic incubation followed 1 h of reperfusion.

### Total RNA extraction and miRNA microarray

Total RNA was extracted using the mirVana miRNA isolation kit (Thermo Fisher Scientific) for cultured cells and brain tissues according to the manufacturer’s protocol. RNA integrity for each sample was confirmed with the Agilent 2100 Bioanalyzer (Agilent Technologies, Santa Clara, CA, USA).

miRNA expression profiling was determined using the Agilent 8 × 60 K mouse miRNA microarray (Agilent Technologies) according to the manufacturer’s instruction. The arrays were scanned using the Agilent Technologies G2600D SG12494263 (Agilent Technologies) to obtain the hybridization images. The array data export processing and analysis was performed using Agilent Feature Extraction v11.0.1.1 (Agilent Technologies).

To identify differentially expressed miRNAs (DEmiRs), we applied moderated t-statistics based on an empirical Bayesian approach^14^. Significantly upregulated and downregulated miRNAs were described as differentially expressed if the *p*-values were < 0.05, and the fold change was greater than 1.5 or less than 0.75. Finally, we excluded miRNAs with a low expression level (average log_2_ expression level < 4.0) from the list of DEmiRs.

### Reverse transcription-quantitative polymerase chain reaction (RT-qPCR)

One microgram of total RNA was converted to complementary DNA (cDNA) using Superscript II reverse transcriptase (Thermo Fisher Scientific) and oligo-(dT)^12–18^ primers (Thermo Fisher Scientific) according to the manufacturer’s instructions. qPCR was performed in a 20-μl reaction mixture containing 1 μl cDNA, 10 μl SYBR Premix EX Taq (Takara Bio, Otsu, Japan), 0.4 μl Rox reference dye (50x, Takara Bio), and 200 nM primers for each gene. The primer sequences of three mouse genes (*B-cell lymphoma 2, Bcl2; Bcl2 associated X, Bax; glyceraldehyde 3-phosphate dehydrogenase, GAPDH*) were: *Bcl2* (forward), 5′-GTCGCT ACCGTCGTGACTTC -3′; *Bcl2* (reverse), 5′-CAGACATGCACCTACCCAGC -3′; *Bax* (forward), 5′-TGAAGACAGGGGCCTTTTTG -3′; *Bax* (reverse), 5′-AATTCGCCGGAG ACACTCG -3′; *GAPDH* (forward), 5′-AATGTGTCCGTCGTGGATCT -3′; and *GAPDH* (reverse), 5′-GGTCCTCAGTGTAGCCCAAG -3′. The reactions were run on a 7500 fast Real-Time PCR system (Thermo Fisher Scientific) at 95 °C for 30 s, followed by 40 cycles of 95 °C for 3 s and 60 °C for 30 s, and a single dissociation cycle of 95 °C for 15 s, 60 °C for 60 s, and 95 °C for 15 s. All PCR reactions were performed in triplicate, and the specificity of the reaction was detected by melting-curve analysis at the dissociation stage. Comparative quantification of each target gene was performed based on cycle threshold (C_T_) normalized to *GAPDH* using the 2^−ΔΔCt^ method.

Mature miRNA quantification was performed using TaqMan MicroRNA Assays for mmu-miR-33-5p, mmu-miR-135b-5p, mmu-miR-551b-3p and snoRNA 202 according to manufacturer recommended protocols (Thermo Fisher Scientific). snoRNA 202 was used as endogenous control. Ten nanograms of total RNA, 50 nM stem-loop RT primer, RT buffer, 0.25 mM each dNTP, 3.33 units/ml MultiScribe reverse transcriptase, and 0.25 units/ml RNase inhibitor were used in 15 μl RT reactions for 30 min at 16 °C, 30 min at 42 °C, and 5 min at 85 °C, using the TaqMan MicroRNA reverse transcription kit (Thermo Fisher Scientific). For qPCR, 1.33 μl (1:15 dilution) of cDNA, 0.2 mM TaqMan probe, 1.5 mM forward primer, 0.7 mM reverse primer, and TaqMan Universal PCR Master Mix (Thermo Fisher Scientific) were added in 20 μl reactions for 10 min at 95 °C and 40 cycles of 15 s at 95 °C and 1 min at 60 °C using a 7500 fast Real-Time PCR system (Thermo Fisher Scientific).

### Transfections

mirVana miRNA mimics and miRNA inhibitors for mmu-miR-33-5p, mmu-miR-135b-5p and negative controls were obtained from Thermo Fisher Scientific. Neuro-2a cells were seeded into plates 18–24 h before transfection. Transfection experiments were performed using 100 nM miRNA mimics or miRNA inhibitors and Lipofectamine 2000 (Thermo Fisher Scientific), according to the manufacturer’s instructions. After 24 h transfection, the cells were subjected to 5 h hypoxia and 1 h reperfusion.

To establish a transient expression system of ABCA1, neuro-2a cells were transfected with mABCA1 expression construct Ex-Mm30260-M10 (GeneCopoeia, Rockville, MD, USA) or pEGFP-N3 (Clontech, Mountain View, CA, USA) plasmids using Metafectene pro transfection reagent (Biotex, Munich, Germany) according to the manufacturer’s protocol. Overexpression of *mAbca1* was confirmed using RT-qPCR 24 h post-transfection.

Pre-designed small interfering RNA (siRNA) for m*Abca1*(siAbca1, CAT#ID L-040248-01-0005) and a non-targeting control (siNC, CAT#ID D-001206-13-05) were purchased from Thermo Fisher Scientific. To deplete *mAbca1*, Neuro-2a cells were transfected with 100 nM siAbca1 or siNC using Metafectene pro transfection reagent (Biotex) according to the manufacturer’s manual. Knockdown of *mAbca1* was confirmed using RT-qPCR 24 h post-transfection.

For OGD, the cells were detached from the plates after 24 h transfection and reseeded in a 35 mm culture dish at a density of 5 × 10^5^ cells/ml. After 24 h reseeding, the cells were washed with phosphate-buffered saline and replaced with glucose-free RKRB buffer. The cells were then placed in hypoxia chamber (Astec) for 8 h at 5% CO_2_, 1% O_2_ and 94% N_2_. After hypoxic treatment, Neuro-2a cells were returned normoxic condition with normal culture media for 1 h for reperfusion.

### Cell viability assay

Cell viability assays were performed using Cell Counting Kit-8 (CCK-8; Dojindo Molecular Technologies, Inc., Kumamoto, Japan) according to the manufacturer’s instructions. The absorbance at 450 nm was measured using a microplate reader (Molecular Devices LLC, Sunnyvale, CA, USA) after incubation for 2 h at 37 °C. Cell viability was expressed as a percentage of that of the control cells.

### miRNA target prediction and dual luciferase reporter assay

To identify the potential target genes for mmu-miR-33-5p and mmu-miR-135b-5p, we used 3 different target prediction programs: miRDB (http://mirdb.org), MIRTarBase (http://mirtarbase.mbc.nctu.edu.tw/) and miRanda (http://microrna.org/).

A fragment of the 3’-untranslated region (UTR) of *ATP-binding cassette, sub-family A, member1* (*Abca1*) contains putative binding regions of mmu-miR-33-5p and mmu-miR-135b-5p (7194–8118, NM_013454.3) was cloned into the *Xho* I and *Not* I sites of the psiCHECK2 Vector (Promega, Madison, WI, USA).

The psiCHECK2-Abca1-3’UTR and miR-33-5p/miR-135b-5p mimic or miRNA mimic negative control were co-transfected into Neuro-2a cells using Lipofectamine 2000 (Thermo Fisher Scientific), according to the manufacturer’s instructions. After 48 h transfection, reporter activity was measured using a dual luciferase reporter gene assay kit (Promega).

### Western blot analyses

The ipsilateral cortex and whole cell lysate were prepared in RIPA buffer (Thermo Scientific) supplemented with protease inhibitors (Thermo Scientific). Proteins (50 μg) were resolved using denaturing 6% sodium dodecyl sulfate–polyacrylamide gel electrophoresis (SDS-PAGE) and transferred to polyvinylidene fluoride (PVDF) membranes. Membranes were blocked in 5% skim milk in Tris-buffered saline with 0.1% Tween 20 (TBST) and subsequently incubated overnight at 4 °C with the following primary antibodies: anti-ABCA1 monoclonal antibody (1:2000, R&D Systems, Minneapolis, MN, USA), anti-caspase-3 monoclonal antibody (1:200, Santa Cruz Biotechnology, Dallas, TX, USA), anti-caspase-8 monoclonal antibody (1:200, Santa Cruz Biotechnology), anti-caspase-9 monoclonal antibody (1:200, Santa Cruz Biotechnology), anti-BCL2 monoclonal antibody (1:1000, Cell Signalling Technology, Danvers, MA, USA), and anti-α-tubulin monoclonal antibody (1:1000, Cell Signalling Technology, Danvers, MA, USA). After washing, the membranes were incubated with secondary antibodies conjugated to horseradish peroxidase for 1 h at room temperature. Chemiluminescence was detected using Super Signal West Dura substrate (Thermo Scientific) according to the manufacturer's protocol. Bands were visualized using a Luminescent Image analyser LAS-300 (General Electric, Uppsala, Sweden) and quantified using Image Gauge software (*Fuji Photo Film*, Tokyo, *Japan*).

### Mitochodrial membrane permeability assay

Changes in cellular mitochondrial membrane potential (MMP, Δψm) were determined by staining the cells with a fluorescent cationic dye, 5,5′,6,6′-tetrachloro-1,1′,3,3′-tetraethyl-benzamidazolocarbocyanin iodide (JC-1) using Mit-E-Ψ Mitochondrial Permeability Detection Kit (Enzo Life Sciences, Inc., New York,USA) according to the manufacturer’s instructions prior to flow cytometry analysis (FACSCalibur; Becton Dickinson, Franklin Lakes, NJ, USA). Data analysis was performed with CellQuest software (Becton Dickinson) by measuring both the green (530 nm, FL-1) and red (585 nm, FL-2) JC-1 fluorescence. At least 10,000 events were collected per sample.

### Microarray data collection

Stroke-related human blood mRNA expression data (GSE16561 and GSE58294) were collected from the Gene Expression Omnibus (GEO, http://www.ncbi.nlm.nih.gov/geo) datasets. The GSE16561 dataset included 39 patients and 24 controls. Each sample was tested using the Illumina HumanRef-8 v3.0 Expression BeadChip (*Illumina*, San Diego, CA, *USA*). The GSE58294 dataset contained 69 ischemic stroke samples and 23 controls. The ischemic stroke samples collected at 3 different time point within 3 h (prior to treatment), 5 h and 24 h (after treatment) after stroke onset (n = 23). All 92 samples were tested using the Affymetrix Human Genome U133 Plus 2.0 Array (Affymetrix, Santa Clara, CA, USA).

### Statistical analysis

All data are expressed as the mean ± standard deviation of at least three independent experiments. Statistical analyses were carried out using GraphPad Prism 5 software. The details of each statistical analysis are provided in the figure legends. We considered *p*-values < 0.05 to be statistically significant.

## Results

### Effects of IPC on apoptosis-associated gene expression in the ischemic cortex

**Figure 1:**
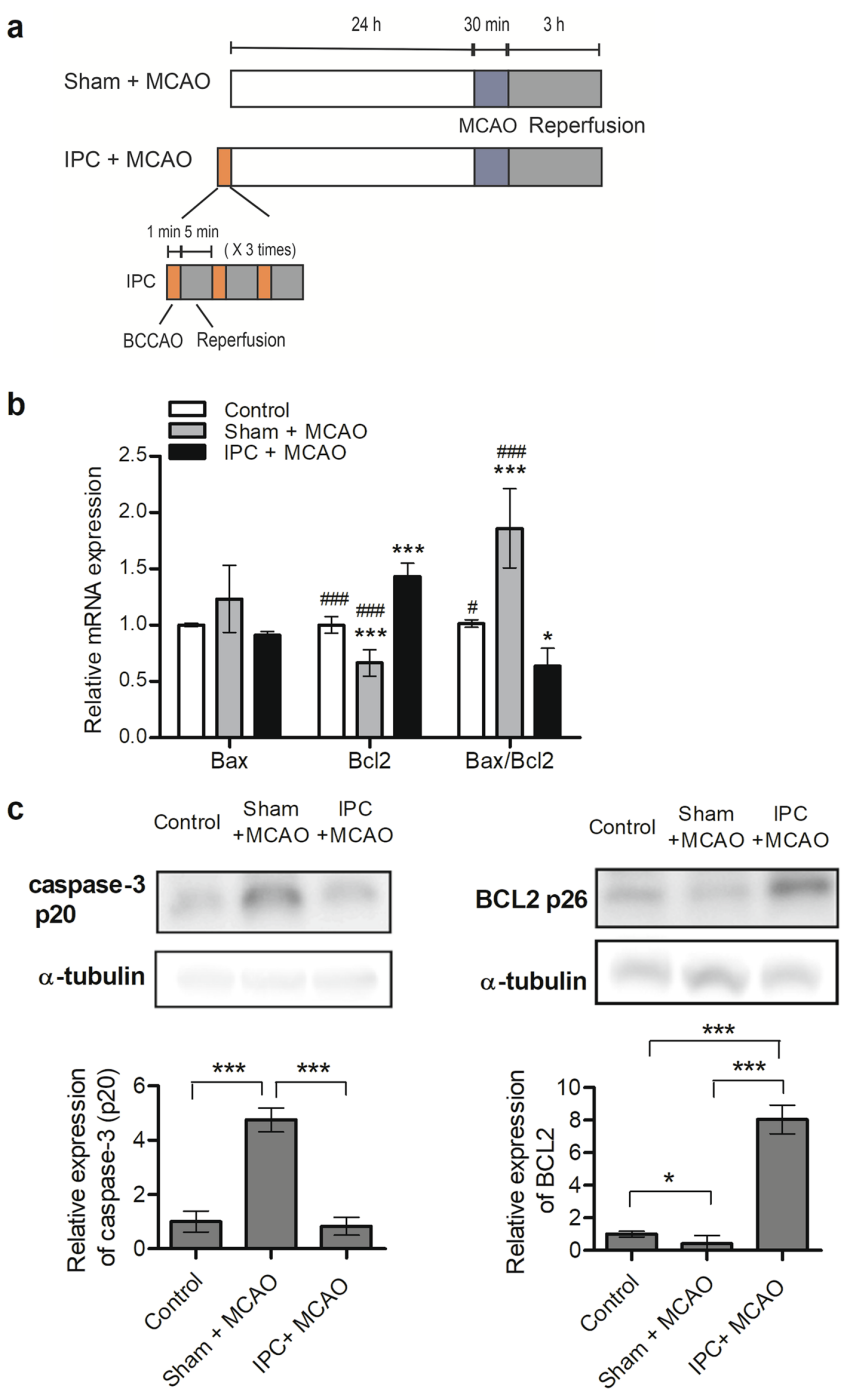
Timeline of experiments and changes to Bax/Bcl2 ratios in ischemic cortex. (**a**) Experimental timeline of Sham + MCAO and IPC + MCAO. IPC was induced by three episodes of 1 min BCCAO with 5 min intervals (n = 6). (**b**) Three hours after reperfusion, the ipsilateral cortex was harvested from MCAO-treated mouse brain, and total RNA was extracted. Bax and Bcl2 mRNA expression was determined by RT-qPCR. Data are represented as mean ± standard deviation (n = 6). Statistical analyses were performed using a one-way analysis of variance and Dunnett’s multiple comparison post-hoc test for comparing the control group (*) or IPC + MCAO group (#) to the other two groups (* = *p* < 0.05, *** = *p* < 0.001, # = *p* < 0.05, ### = *p* < 0.001). (**c**) Cleaved caspase-3 and BCL2 protein expression was assessed using Western-blot analyses. Values from densitometric analyses are shown after normalization to α-tubulin values from controls. Data are mean ± standard deviation (n = 6). Statistical analyses were performed using *t*-tests (* = *p* < 0.05, *** = *p* < 0.001). Full-length blots are presented in Supplementary Fig. 1. Abbreviations: Bax, Bcl2 associated X; Bcl2, B-cell lymphoma 2; MCAO, transient middle cerebral artery occlusion; IPC, ischemic preconditioning; BCCAO, bilateral common carotid artery occlusion; RNA, ribonucleic acid; mRNA, messenger ribonucleic acid; RT-qPCR, reverse transcription-quantitative polymerase chain reaction.

The experimental timelines for sham + MCAO and IPC + MCAO are shown in Fig. 1a. We previously reported ischemic protection of the BCCAO IPC paradigm by showing a smaller infarct volume (~ 48%) in the IPC + MCAO group compared to the control and sham + MCAO groups. Ischemic injury reduction was observed predominantly in the cortex but not in the stratum^11^. Previous studies using animal models of brain ischemia reported neuroprotective effects of IPC through inhibition of apoptosis^15,16^. Therefore, we used RT-qPCR to compare the expression of apoptosis-promoting *Bax* mRNA and apoptosis-inhibiting *Bcl2* mRNA in the IPC + MCAO group (n = 6) to their expressions in the control group (n = 6) and the sham + MCAO group (n = 6). There was no difference in *Bax* mRNA expression among the three groups; however, the IPC group showed significantly higher *Bcl2* mRNA expression compared to both the control and sham + MCAO groups (Fig. 1b). In addition, the IPC + MCAO *Bax*/*Bcl2* mRNA ratio was decreased significantly to approximately 37% and 123% of control and sham + MCAO group ratios, respectively, indicating a neuroprotective effect of IPC against apoptosis (Fig. 1b). Neuronal apoptotic reduction after preconditioning was further evaluated by determining protein expression of cleaved caspase-3 and anti-apoptotic BCL2 in the control (n = 6), sham + MCAO (n = 6), and IPC + MCAO groups (n = 6). Cleaved caspase-3 protein expression was significantly reduced, approximately 5.9-fold, in the IPC + MCAO group compared to the sham + MCAO group, whereas anti-apoptotic BCL2 protein expression was significantly increased, approximately 19.1-fold, in the IPC + MCAO group compared to the sham + MCAO group (Fig. 1c).

### Identification of IPC-specific miRNAs

**Figure 2:**
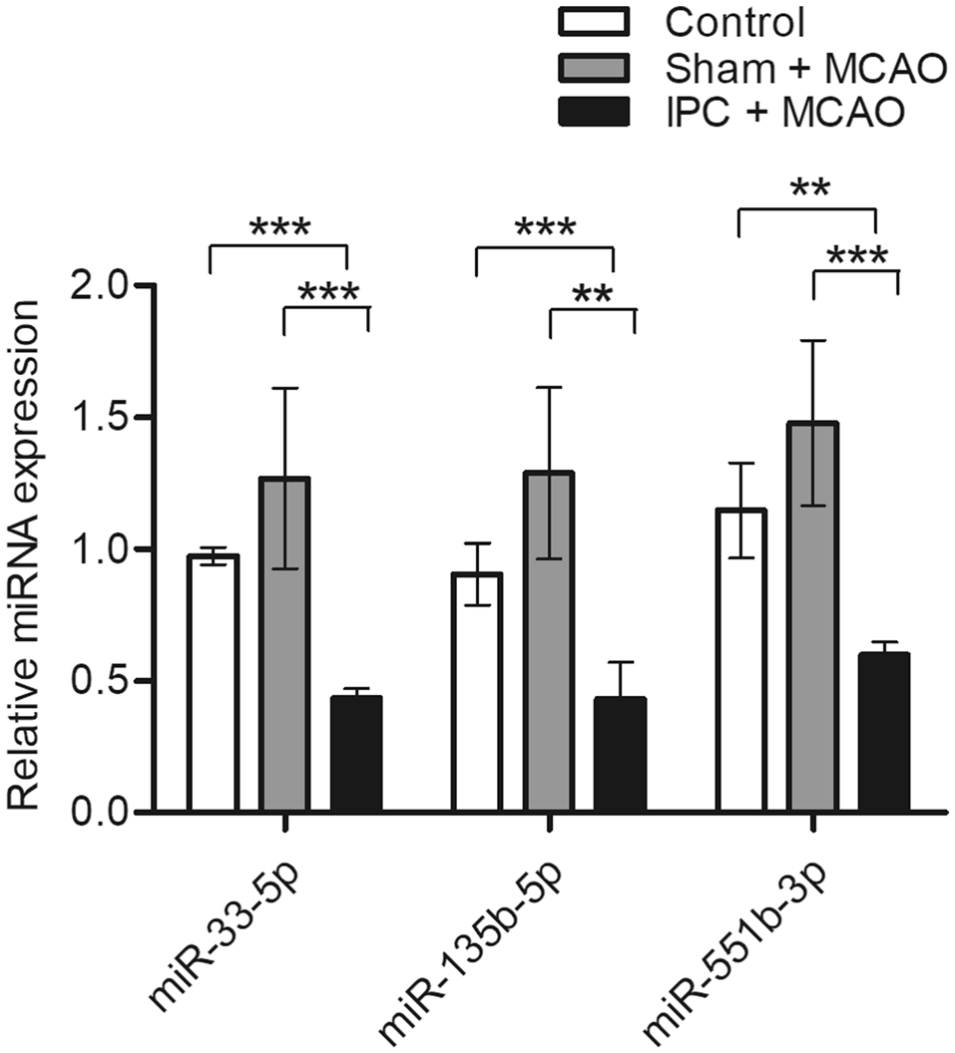
Downregulation of selected miRNAs after IPC. Expression patterns of selected miRNAs from the ipsilateral cortex were validated by RT-qPCR at 3 h after reperfusion and MCAO in both sham and preconditioned mice. Data are shown as mean ± standard deviation (n = 6). Statistical analyses were performed using t-tests (** = *p* < 0.01, *** = *p* < 0.001). Abbreviations: miRNA, micro ribonucleic acid; IPC, ischemic preconditioning; RT-qPCR, reverse transcription-quantitative polymerase chain reaction; MCAO, transient middle cerebral artery occlusion.

To identify neuroprotection-associated changes in miRNA expression in the ipsilateral cortex after ischemic stroke, we compared the miRNA expression profile of the IPC + MCAO (n = 5) group with the sham + MCAO (n = 5) group. DEmiRs were then selected using the following criteria: upregulated (fold change > 1.5, *p* < 0.05, average log_2_ expression level < 4.0) or downregulated (fold change < 0.75, *p* < 0.05, average log_2_ expression level < 4.0). We identified five candidate DEmiRs using these criteria (Table 1). Because the neuroprotective effects of the miR-200 family (mmu-miR-200a-3p and mmu-miR-200b-3p) in IPC have been previously reported^7^, we focused on the three remaining miRNAs: mmu-miR-33-5p, mmu-miR-135b-5p, and mmu-miR-551b-3p. Downregulation of these three miRNAs was verified using RT-qPCR (Fig. 2), which showed that all were significantly decreased in the IPC + MCAO group (n = 6) compared to the sham + MCAO group (n = 6) and the control group (n = 6).

**Table 1:** Identification of five IPC-associated miRNAs.

miRNA	Regulation	Fold change^a^	*p*-value^b^	Average log_2_ expression level
mmu-miR-200a-3p	Upregulation	4.64	0.0209	6.44
mmu-miR-200b-3p	Upregulation	4.68	0.0457	6.67
mmu-miR-33-5p	Downregulation	0.64	0.0363	5.90
mmu-miR-135b-5p	Downregulation	0.59	0.0120	4.02
mmu-miR-551b-3p	Downregulation	0.52	0.0484	9.68

miRNA, micro ribonucleic acid; IPC, ischemic preconditioning; BCCAO, bilateral common carotid artery occlusion; MCAO, transient middle cerebral artery occlusion.

^a^Fold changes were calculated as the ratio of BCCAO + MCAO (n = 5) to Sham + MCAO (n = 5).

^b^Statistical analysis was performed using a t-test.

### Neuroprotective effects by inhibition of miR-33-5p and miR-135b-5p under in vitro ischemic conditions

**Figure 3:**
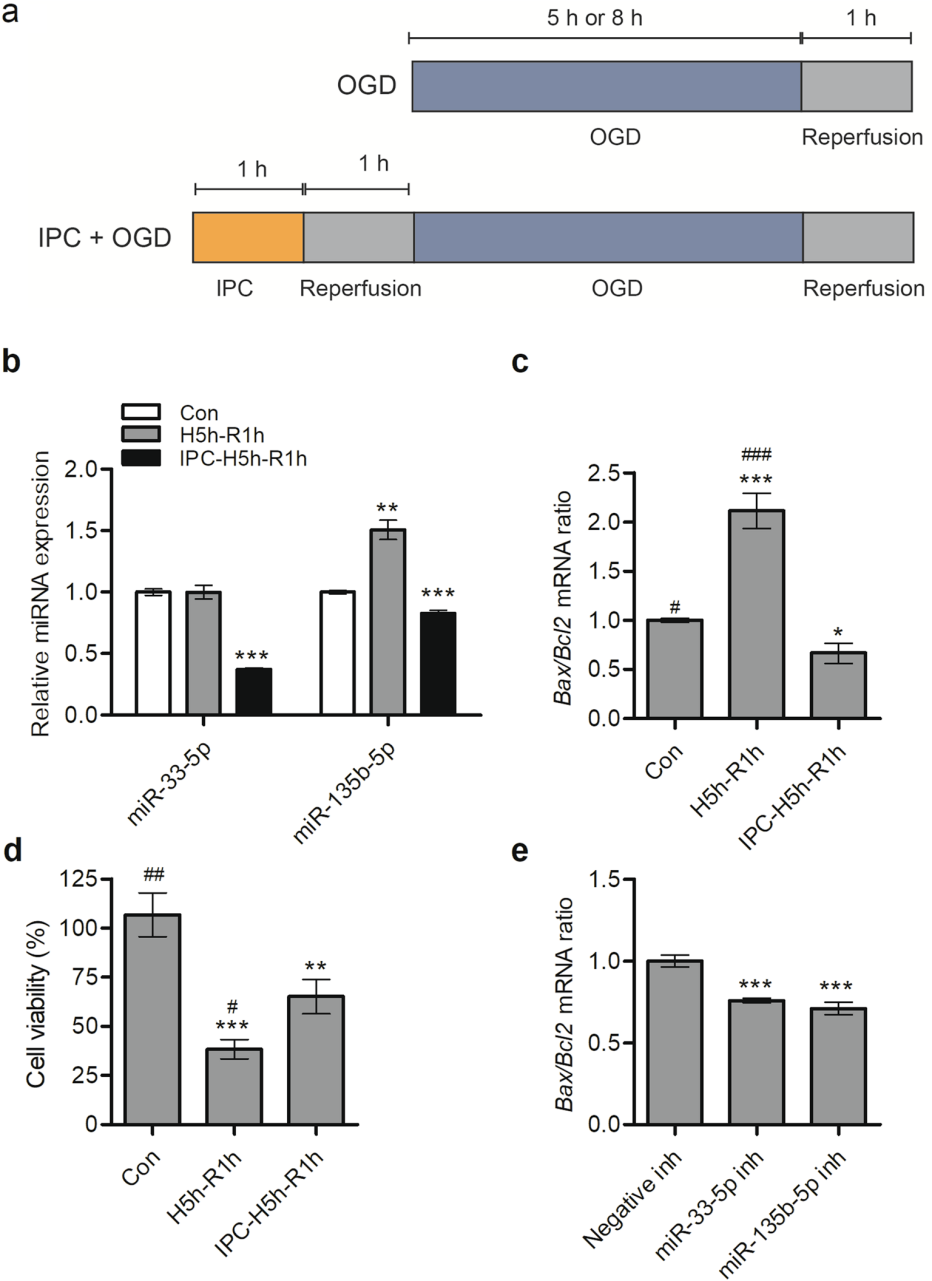
Neuroprotective effects by downregulation of miR-33-5p and miR-135b-5p. (**a**) Experimental timeline for treatment of OGD and IPC + OGD in Neuro-2a cells. (**b**) After treatment of OGD or IPC + OGD, expression levels of two miRNAs (miR-33-5p and miR-135b-5p) were determined using RT-qPCR. (**c**) After treatment of OGD or IPC + OGD, mRNA levels of pro-apoptotic *Bax* and anti-apoptotic *Bcl2* were determined by RT-qPCR and expressed as *Bax/Bcl2* mRNA ratios. (**d**) After treatment of OGD or IPC + OGD, cell viability was determined by CCK-8 assay. (**e**) To suppress the function of each miRNA, Neuro-2a cells were transfected with either miR-33-5p inhibitor, miR-135b-5p inhibitor, or a relevant negative-control inhibitor. Twenty-four hours after transfection, cells were subjected to OGD. Expression of pro-apoptotic *Bax* and anti-apoptotic *Bcl2* mRNA were determined by RT-qPCR and expressed as *Bax/Bcl2* mRNA ratios. Values are mean ± standard deviation for n = 3. Statistical analyses were performed using one-way analysis of variance followed by Dunnett’s multiple comparison test using control (*), negative inhibitor (*), or IPC + OGD (#) (* = *p* < 0.05, ** = *p* < 0.01, *** = *p* < 0.001, # = *p* < 0.05, ## = *p* < 0.01, ### = *p* < 0.001). Abbreviations: OGD, oxygen–glucose deprivation; IPC, ischemic preconditioning; miRNA, micro ribonucleic acid; RT-qPCR, reverse transcription-quantitative polymerase chain reaction; Bax, Bcl2 associated X; Bcl2, B-cell lymphoma 2; mRNA, messenger ribonucleic acid; CCK-8, cell counting kit-8; H5h, hypoxic incubation for 5 h; R1h, reperfusion for 1 h; inh, inhibitor.

To mimic in vitro ischemic-like conditions, Neuro-2a cells were exposed to OGD, following the experimental timeline shown in Fig. 3a for OGD and IPC + OGD. Expression levels of the three miRNAs (miR-33-5p, miR-135b-5p, and miR-551b-3p) were determined under in vitro ischemic conditions using RT-qPCR. The expression of miR-551b-3p was too low to be detected in Neuro-2a cells; however, consistent with the results of our ischemic-stroke mouse model, the expression of both miR-33-5p and miR-135b-5p were significantly downregulated by in vitro IPC stimulation compared to the control or in vitro ischemic-condition levels (Fig. 3b). Furthermore, in vitro IPC stimulation significantly decreased the *Bax*/*Bcl2* mRNA ratios by approximately 169% and 34% compared to the ratios for ischemic conditions and control conditions, respectively (Fig. 3c). Cell viability was significantly increased by in vitro IPC stimulation compared to that of in vitro ischemic-conditions (Fig. 3d). To determine whether downregulation of miR-33-5p and miR-135b-5p protected Neuro-2a cells from ischemia-induced apoptosis, cells were transfected with individual miRNA inhibitors and then assessed for apoptosis after ischemia treatment. *Bax*/*Bcl2* mRNA ratios were significantly reduced in cells transfected with miR-33-5p and miR-135b-5p inhibitors, approximately 24% and 29%, respectively, compared to the ratio in cells transfected with negative-control inhibitor (Fig. 3e). Expression inhibition by both miR-33-5p and miR-135b-5p showed potent protection against ischemia-induced apoptosis.

### miRNA-33-5p and miRNA-135b-5p negatively regulate ABCA1 expression

**Figure 4:**
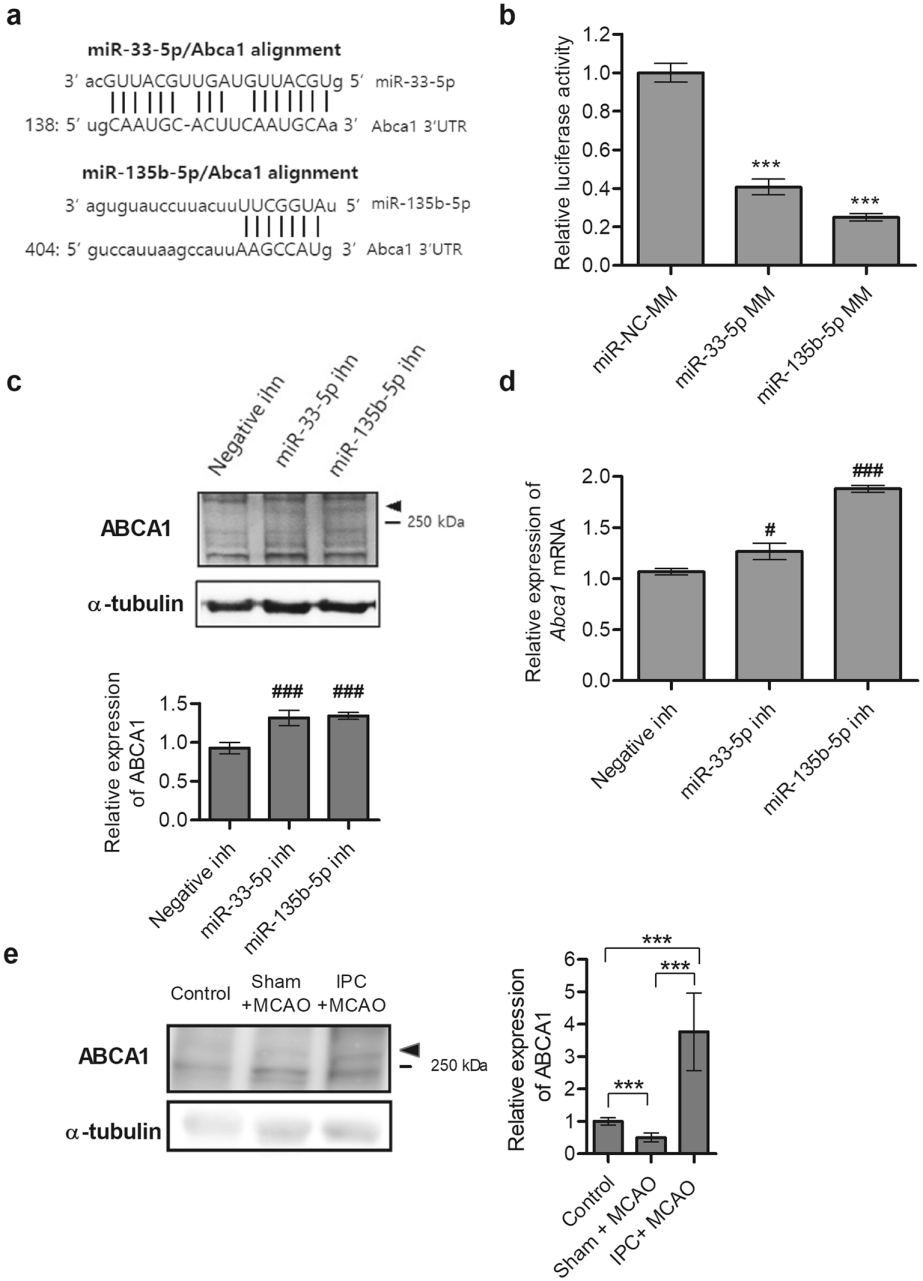
Identification of *Abca1* mRNA as a target for miR-33-5p and miR-135b-5p. (**a**) The 3’-UTR of ATP-binding cassette subfamily A member1 (*Abca1*) mRNA contains putative binding regions for miR-33-5p and miR-135b-5p. (**b**) The relative luciferase activity of an *Abca1* 3′-UTR reporter plasmid was assayed in Neuro-2a cells after transfection with miR-33-5p mimic (MM), miR-135b-5p MM, or miRNA negative-control mimic (miR-NC-MM). (**c**) Neuro-2a cells were transfected with miR-33-5p inhibitor, miR-135b-5p inhibitor, or a relevant negative-control inhibitor. Twenty-four hours after transfection, cells were subjected to 5 h of hypoxia and 1 h of reperfusion. ABCA1 protein expression was then assessed using Western-blot analysis. Representative results are illustrated, and values from densitometric analyses are shown after normalization to α-tubulin from the controls. Full-length blots are presented in Supplementary Fig. 2. (**d**) *Abca1* mRNA expression was measured by RT-qPCR. Data are represented as mean ± standard deviation from three experiments. Statistical analyses were performed using one-way analysis of variance followed by Dunnett’s multiple comparison test using negative inhibitor as the control (# = *p* < 0.05, ### = *p* < 0.001). (**e**) MCAO for 30 min was performed in C57BL/6 mice 24 h after either a sham or IPC operation. After 3 h of reperfusion, the ipsilateral cortex was harvested and ABCA1 protein expression was assessed using Western-blot analysis. Values from densitometric analyses are shown after normalization to α-tubulin values from the controls. Data are mean ± standard deviation (n = 6). Statistical analyses were performed using *t*-tests (* = *p* < 0.05, ** = *p* < 0.01, *** = *p* < 0.001). Full-length blots are presented in Supplementary Fig. 3. Abbreviations: UTR, untranslated region; miR, micro ribonucleic acid; inh, inhibitor; MCAO, transient middle cerebral artery occlusion; IPC, ischemic preconditioning.

To explore the functions of miR-33-5p and miR-135b-5p under ischemic conditions, their putative gene targets were identified by miRDB (http://mirdb.org), MirTarBase (http://mirtarbase.mbc.nctu.edu.tw/), and miRanda (http://microrna.org/). The 3’-untranslated region (UTR) of *Abca1* was identified as a target for both miR-33-5p and miR-135b-5p by miRanda (Fig. 4a). To determine if miR-33-5p and miR-135b-5p directly regulate the *Abca1* target by binding the 3’-UTR, we cloned the predicted target site (nucleotides 7194–8118) of *Abca1* mRNA downstream of a luciferase reporter gene and co-transfected this construct together with miR-33-5p/miR-135b-5p mimic or miRNA negative-control mimic into Neuro-2a cells. Luciferase activity decreased approximately 64% and 77% in cells transfected with miR-33-5p mimic or miR-135b-5p mimic, respectively, compared to the negative control (Fig. 4b). To further test our hypothesis that miR-33-5p and miR-135b-5p directly regulate protein expression, we assessed ABCA1 expression under hypoxic conditions after treatment with each miRNA inhibitor. Knockdown of miR-33-5p and miR-135b-5p significantly upregulated ABCA1 protein expression (Fig. 4c) and *Abca1* mRNA expression (Fig. 4d). These results provide strong evidence that *Abca1* mRNA is a target for miR-33-5p and miR-135b-5p, negatively regulating ABCA1 expression.

As shown in Fig. 2, miR-33-5p and miR-135b-5p levels were downregulated in the IPC + MCAO group compared to the control and sham + MCAO group. To confirm the role of miR-33-5p and miR-135b-5p in negatively regulating target protein expression in vivo, ABCA1 protein expression was determined using Western-blot analysis in the control (n = 6), sham + MCAO group (n = 6), and IPC + MCAO group (n = 6). Protein expression was significantly higher in the IPC + MCAO group compared to the control and sham + MCAO group (Fig. 4e).

### ABCA1 prevents OGD-induced mitochondria-dependent apoptotic signaling

**Figure 5:**
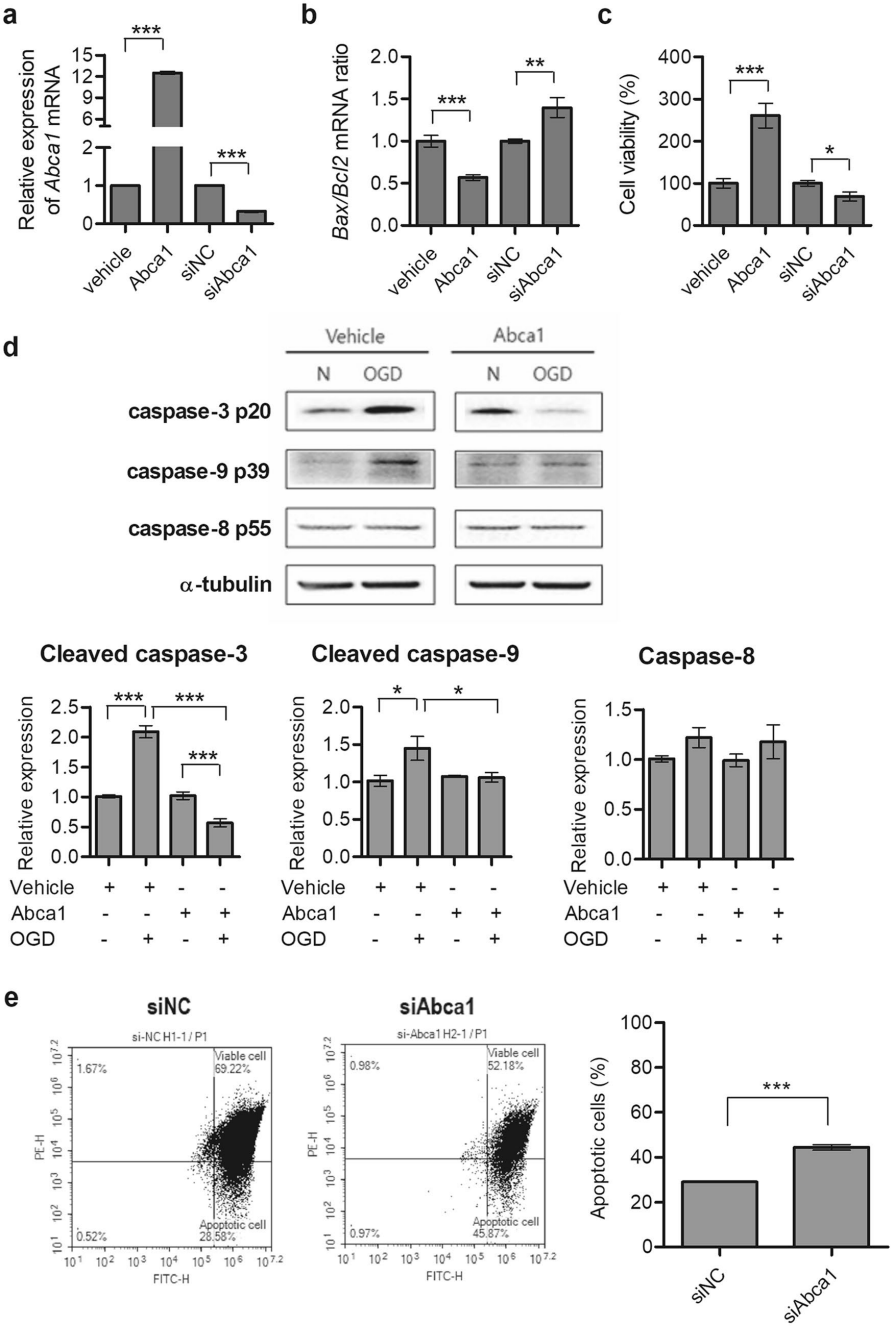
ABCA1 suppresses a mitochondria-dependent apoptosis pathway. (**a**–**c**) Neuro-2a cells were transiently transfected with either vehicle or *Abca1*, siNC, and siAbca1 expression constructs. For OGD, cells were detached from plates 24 h after transfection and reseeded. Twenty-four hours after reseeding, cells were subjected to 8 h of OGD and 1 h of reperfusion. mRNA expression of *Abca1*, *Bax*, and *Bcl2* was measured by RT-qPCR (n = 3). Cell viability was determined by CCK-8 assay. (**d**) Caspase-3, -8, and -9 activation was assessed by Western-blot analysis. Representative results are illustrated, and values from densitometric analyses are shown after normalization to α-tubulin from the controls (n = 3). Full-length blots are presented in Supplementary Fig. 4 (**e**) Mitochondrial membrane potentials were determined by flow cytometry analysis using JC-1 staining. Representative results are illustrated, and values are reported mean ± standard deviation (n = 4). Statistical analyses were performed using *t*-tests (* = *p* < 0.05, ** = *p* < 0.01, *** = *p* < 0.001). Abbreviations: Abca1, ATP-binding cassette subfamily A member 1; siNC, non-targeting control small interfering ribonucleic acid; siAbca1, Abca1 siRNA; OGD, oxygen–glucose deprivation; mRNA, messenger ribonucleic acid; RT-qPCR, reverse transcription-quantitative polymerase chain reaction; Bax, Bcl2 associated X; Bcl2, B-cell lymphoma 2; JC-1, 5,5′,6,6′-tetrachloro-1,1′,3,3′-tetraethyl-benzamidazolocarbocyanin iodide.

To determine the effect of ABCA1 on OGD-induced neuronal apoptosis, Neuro-2a cells were transiently transfected with *Abca1* expression constructs or with *Abca1* siRNA (siAbca1) to overexpress or knockdown ABCA1, respectively; cells transfected with a vehicle plasmid construct or non-targeting siRNA (siNC) were used as controls. After an additional 24 h, cells were subjected to 8 h of hypoxia with glucose-free RKRB buffer and reperfusion for 1 h with normal culture media. Following OGD, *Abca1* expression levels were assessed using RT-qPCR: *Abca1* mRNA increased ~ 12-fold in *Abca1*-transfected cells and decreased ~ 70% in siAbca1-transfected cells compared to mock-transfected cells (Fig. 5a). Compared to controls, *Bax*/*Bcl2* mRNA ratios were significantly decreased ~ 47% in *Abca1* overexpressed cells and significantly increased ~ 52% in *Abca1* knockdown cells following OGD (Fig. 5b), and cell viability was significantly increased ~ 160% in *Abca1* overexpressed cells but decreased ~ 31% in *Abca1* knockdown cells compared to their controls (Fig. 5c). Caspases are crucial mediators of cellular apoptosis, so we investigated the influence of ABCA1 on caspase -3, -8, and -9 activation following OGD using Western-blot analysis. We found that OGD-induced activation of caspase-3 and -9 was significantly inhibited approximately 75% and 50%, respectively, in *Abca1*-transfected cells compared to mock-transfected cells, but caspase-8 activity was unchanged (Fig. 5d). In addition, the influence of ABCA1 on MMP was determined using flow cytometry analysis of JC-1 staining. The percentage of cells with reduced mitochondrial membrane potentials (the green fluorescence ratio) increased ~ 20% in siAbca1-transfected cells compared to siNC-transfected cells following OGD, suggesting that ABCA1 attenuated OGD-induced mitochondrial dysfunction (Fig. 5e). These results indicate that ABCA1 protects neuronal cells against OGD-induced apoptosis by inhibiting a mitochondria-dependent apoptosis pathway.

### Upregulation of ABCA1 mRNA expression in blood from stroke patients

**Figure 6:**
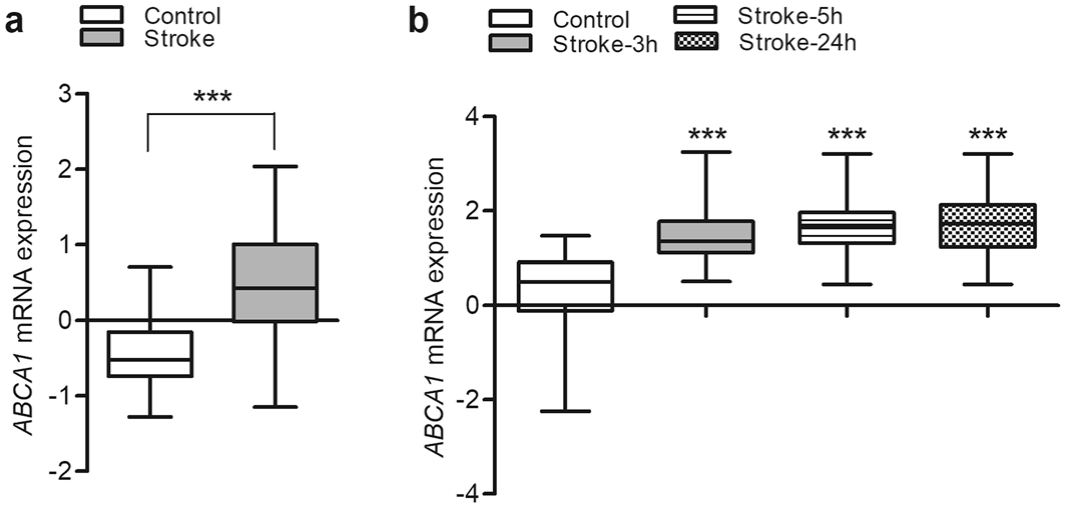
Upregulated *ABCA1* mRNA expression in stroke-patient blood. Human blood mRNA expression levels of *ABCA1* obtained from the Gene Expression Omnibus datasets GSE16561 and GSE58294 are shown in (**a**) and (**b**), respectively. Data are shown as box-and-whisker plots (from minimum to maximum). Statistical analyses were performed using t-tests (**a**, *** = *p* < 0.001) or a one-way analysis of variance followed by Dunnett’s multiple comparison test anchored to control data (**b**, *** = *p* < 0.001). Abbreviations: Abca1, ATP-binding cassette subfamily A member 1; mRNA, messenger ribonucleic acid.

To examine the role of ABCA1 in human stroke patients, mRNA expression data from peripheral blood samples were collected from the following GEO datasets: acute ischemic stroke (GSE16561) and cardioembolic stroke (GSE58294). Database GSE16561 contained transcription expression profiles from 63 blood samples (39 stroke patients and 24 controls); mRNA expression of *ABCA1* was significantly increased in stroke patients compared to controls (Fig. 6a). Database GSE58294 contained transcriptional expression profiles from 92 blood samples (69 stroke patients and 23 controls). The ischemic-stroke samples were collected at three different time points: within 3 h (prior to treatment), 5 h, and 24 h (after treatment) after stroke onset (n = 23). Increased mRNA expression of *ABCA1* was found at all time points in the blood samples from stroke patients compared to controls (Fig. 6b), supporting the idea that ABCA1 expression is induced by brain ischemia. Based on these results, we suggest that the induction of ABCA1 may be a protective response to reduce ischemic brain injury.

## Discussion

Apoptosis caused by cerebral ischemia/reperfusion (I/R) is a very important process for neuronal cell death^17^. The Bcl2 superfamily includes proapoptotic genes such as *Bax* and *Bad*^18^, and anti-apoptotic genes such as *Bcl2*, *Bcl-xL*, and *Bax*^19^. Reduced cellular activation of *Bcl2* and *Bcl-xL*, and increased *Bax* activation has been reported in neurons injured by ischemia^20,21^, supporting the idea that the ratio of proapoptotic/anti-apoptotic proteins can determine cellular fate after a death signal^22^. The present study demonstrates that IPC has a neuroprotective effect on ischemia by modulating the *Bax*/*Bcl2* mRNA ratio. This ratio was significantly decreased in the IPC + MCAO group compared to the control and sham + MCAO groups through increased *Bcl2* mRNA expression without a significant change in *Bax* expression. Bcl2 acts as a survival factor in the mitochondrial apoptotic pathway by inhibiting caspase activation, inhibiting the production of free radicals, and through the actions of other proapoptotic Bcl2 family members such as Bax and Bad^23,24^. Overexpression of Bcl2 also promoted cortical neuronal survival after focal cerebral ischemia by preventing translocation of *apoptosis inducing factor* from mitochondria to the nucleus^25^. Consistent with the present results, cyclic AMP response element binding protein (CREB)-mediated Bcl2 overexpression was also shown to be protective in ischemia-preconditioned rats against subsequent I/R exposure^26^.

To elucidate underlying mechanisms of neuroprotection mediated by IPC, we investigated the miRNA expression profile after IPC + MCAO, and identified neuroprotective expression changes in selected miRNAs, two of which were upregulated (miR-200a-3p and miR-200b-3p) and three of which were downregulated (miR-33-5p, miR-135b-5p and miR-551b-3p) compared to MCAO-only miRNAs in a cerebral ischemia mouse model. Among these, both miR-33-5p and miR-135b-5p targeted ABCA1.

Several studies have investigated IPC-induced miRNA changes using in vivo and in vitro models to elucidate their functional significance in generating tolerance to cerebral ischemic injury. However, due to different experimental designs, different IPC stimuli, and diverse in vivo or in vitro models, the effects of IPC-induced neuroprotection on miRNAs and their targets are highly heterogenous and inconsistent across studies. In one study investigating miRNA changes with preconditioning, the miR-200 and miR-182 families were both upregulated after IPC following 3 h of reperfusion, resulting in neuroprotection through downregulation of prolyl hydroxylase 2 which is involved in the degradation of hypoxia inducible factors^7^. In contrast, another study reported that upregulation of miR-200c after stroke promoted neuronal cell death through inhibiting expression of its target protein (reelin) that plays a role in synaptogenesis and neuronal migration^27^. A subsequent mouse study has shown that decreased miR-132 expression induced neuroprotection by targeting methyl CpG-binding protein 2 following IPC^28^, but miR-132 overexpression in rats using a viral vector has also been reported to increase neuronal survival following global ischemia and decrease cell death following OGD in vitro^29^.

Previous studies have shown that miR-33-5p (human miR-33a-5p) targets key genes involved in cholesterol transport, such as *Abca1* and *Abcg1*. Inhibition of endogenous miR-33-5p in a mouse model increased ABCA1 expression and led to elevated plasma high-density lipoprotein (HDL), reversal of a cholesterol transport pathway, and prevention of atherosclerosis^30,31^. In addition, inhibition of miR-33-5p lowered the endogenous cortical level of amyloid-β in a mouse model of Alzheimer’s disease (AD) via increased ABCA1 expression and APOE lipidation, suggesting a potential therapeutic strategy for AD^32^. Inhibition of miR-33-5p in OGD-treated rat brain microvascular endothelial cells has also been reported to enhance the expression of its other target gene, X-box binding protein 1, improving both survival and angiogenesis through upregulation of the *Differentiation antagonizing non-protein coding RNA* gene^33^. Neuroprotective effects of miR-135b-5p have also been reported in AD and Parkinson’s disease through targeting *β-site APP-cleaving enzyme 1* and *glycogen synthase kinase 3β*, respectively^34,35^. In contrast, the present results demonstrate that inhibition of miR-135b-5p prevented neuronal cell death from OGD-induced apoptosis. This discrepancy is likely the result of a single miRNA targeting different pathways or genes.

In the present study, we demonstrated that both miR-33-5p and miR-135b-5p expression levels were significantly downregulated by IPC, and inhibition of their expression protected neuronal cells from OGD-induced apoptosis (i.e., an in vitro ischemia model). Inhibition of these two miRNAs in neuronal cells significantly increased mRNA and protein levels of ABCA1, and a binding assay showed that these two miRNAs were specific for the 3’UTR of *Abca1* mRNA.

ABCA1 is an integral cell-membrane protein that plays a major role in HDL formation by exporting cholesterol and phospholipids from cells and solubilizing these lipids by apolipoprotein^36^. There is also evidence that ABCA1 has anti-inflammatory activity and other beneficial effects on ischemic injury. Brain evaluation in ABCA1-knockout mice has shown increased blood–brain barrier leakage, white matter/axonal damage, and functional deficits after stroke compared to ABCA1-expressing control mice^37^. ABCA1 overexpression in a mouse stroke model induced by GW3905, a liver-X receptor (LXR) agonist, increased blood HDL, both gray and white matter densities, oligodendrocyte progenitor cell number, and also improved functional outcomes after distal MCAO^38^. In a mouse model of I/R using acute elevation of intraocular pressure, another LXR agonist (TO901317) induced ABCA1 and reduced retinal ganglion cell apoptosis by promoting the nuclear translocation of annexin A1, an anti-inflammatory factor^39^. In the present study, we found that ABCA1 expression was enhanced in both preconditioned ischemic neurons and the brain compared to neurons and brain without preconditioning. We further demonstrated that overexpression of ABCA1 decreased the *Bax/Bcl2* mRNA ratio and activation of caspase-9 and caspase-3, whereas knockdown of ABCA1 expression increased the *Bax/Bcl2* mRNA ratio and the percentage of Neuro-2a cells with MMP loss after OGD-treatment, supporting the idea of a protective role for ABCA1 via apoptosis-pathway blocking in mitochondria. However, the precise molecular mechanism underlying how ABCA1 modulates apoptotic gene expression and MMP under ischemic conditions is not clear and remains to be investigated.

In human blood, the expression of *ABCA1* mRNA was significantly upregulated in stroke patients compared to non-stroke controls, suggesting that increased ABCA1 expression may contribute to cell protection after ischemic injury and prevent further cellular damage.

In conclusion, increased ABCA1 expression following IPC exerts a protective effect against cerebral ischemia via suppression of a mitochondria-dependent apoptosis pathway. The regulatory mechanisms for both miR-33-5p and miR-135b-5p on ABCA1 expression may provide potential therapeutic targets for treating stroke.

## Supplementary Information

Supplementary Information.
