# Supplementary material for: Protective role of ABCA1 in ischemic preconditioning is mediated by downregulation of miR-33-5p and miR-135-5p
Source: Sci Rep. 2021 Jun 15;11:12511. doi: 10.1038/s41598-021-91982-x (PMC8206355; doi:10.1038/s41598-021-91982-x)
Supplement: Supplementary file 1 — Supplementary Information. [file 41598_2021_91982_MOESM1_ESM.docx]

**Protective role of ABCA1 in ischemic preconditioning is mediated by downregulation of miR-33-5p and miR-135-5p**

Hye Youn Sung^a^, Eun Nam Choi^a^, Jihye Han^a^, Yun Ju Chae^a^, Sun-Wha Im^b^, Hee-Sun Kim^c^ Eun-Mi Park^d*^, Jung-Hyuck Ahn^a*^

^a^Department of Biochemistry, College of Medicine, Ewha Womans University, Seoul, Korea

^b^Genomic Medicine Institute, Medical Research Center, Seoul National University, Seoul, Korea

^c^Department of Molecular Medicine, College of Medicine, Ewha Womans University, Seoul, Korea

^d^Department of Pharmacology, College of Medicine, Ewha Womans University, Seoul, Korea

*These authors contributed equally to this work

**Correspondence:**

*Jung-Hyuck Ahn, Address: Department of Biochemistry, College of Medicine, Ewha Womans University, **25 Magokdong-ro 2-gil, Gangseo-gu,** Seoul 07804, Korea Tel: +82-2-6986-6200 Fax: +82-2-6986-7016 E-mail: [ahnj@ewha.ac.kr](mailto:ahnj@ewha.ac.kr)

*Eun-Mi Park, Address: Department of Pharmacology, College of Medicine, Ewha Womans University, **25 Magokdong-ro 2-gil, Gangseo-gu,** Seoul 07804, Korea Tel: +82-2-6986-6170 Fax: +82-2-6986-7017 E-mail: empark@ewha.ac.kr

**Supplementary information**


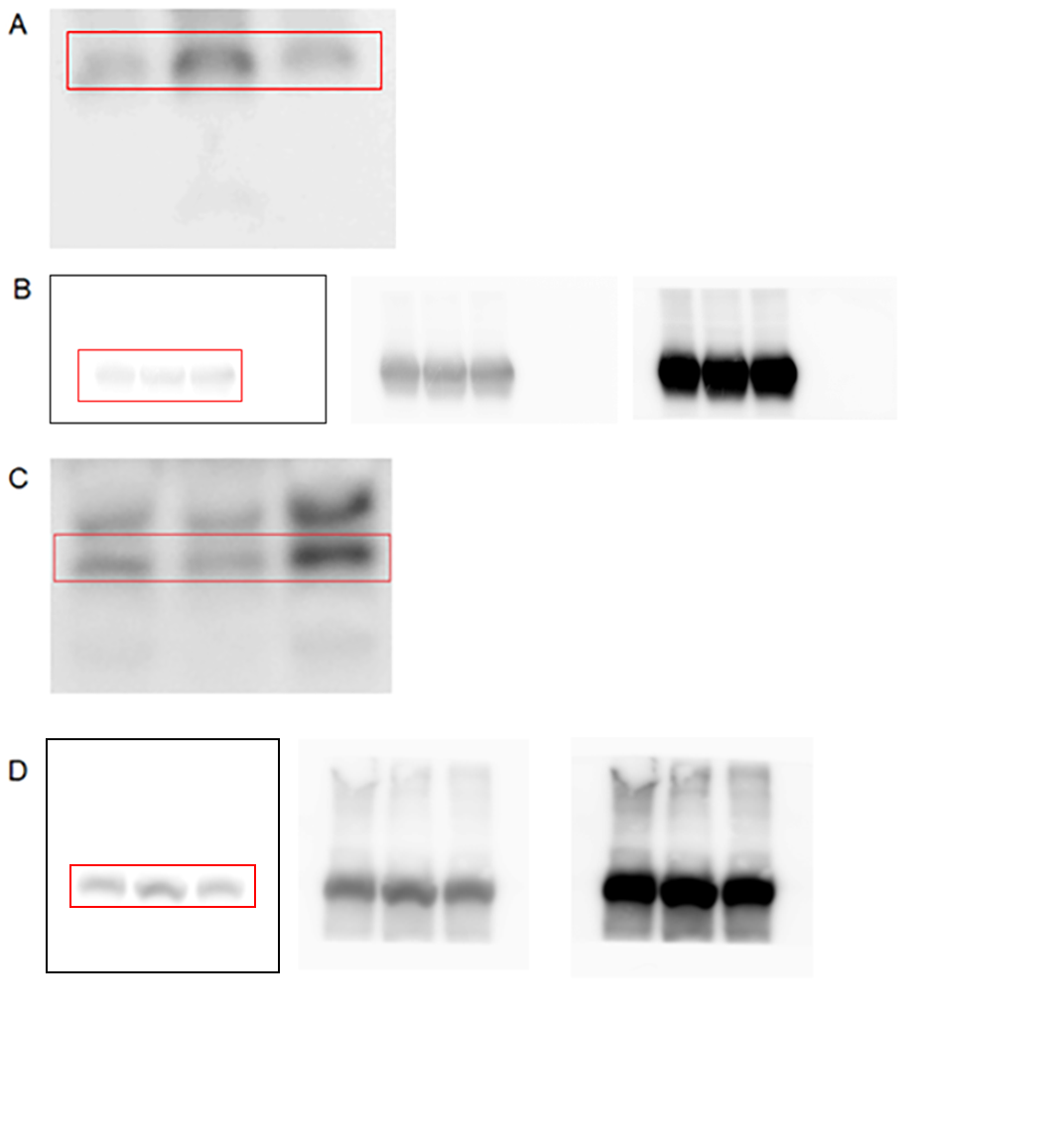


**Supplementary Figure 1**. Original and unprocessed blots of caspase-3 (p20) (**A**), their internal control blots of α-tubulin (multiple exposure images, **B**), BCL2 (p26) (**C**) and their internal control blots of α-tubulin (multiple exposure images, **D**) western blot analysis from Figure 1c. Showed blots at Figure 1c are marked with red boxes.

**
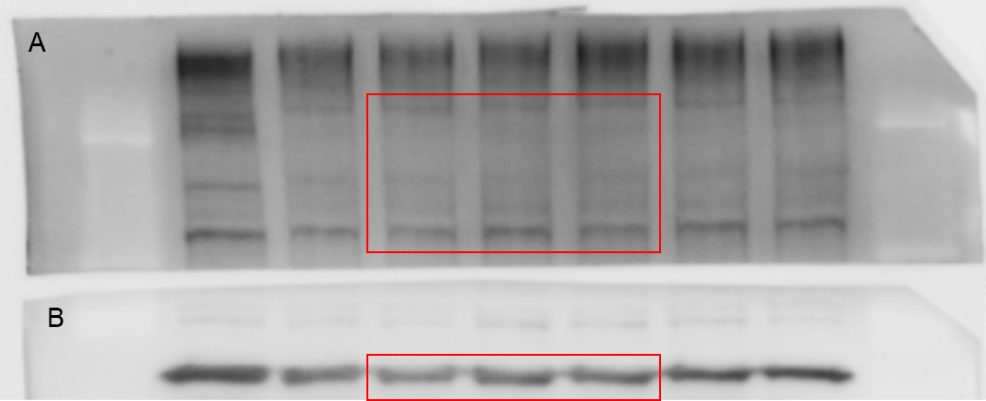
**

**Supplementary Figure 2**. Original and unprocessed blots of ABCA1western blot analysis (**A**) from Figure 4c and their internal control blots of α-tubulin (**B**). Showed blots at Figure 4c are marked with red boxes.


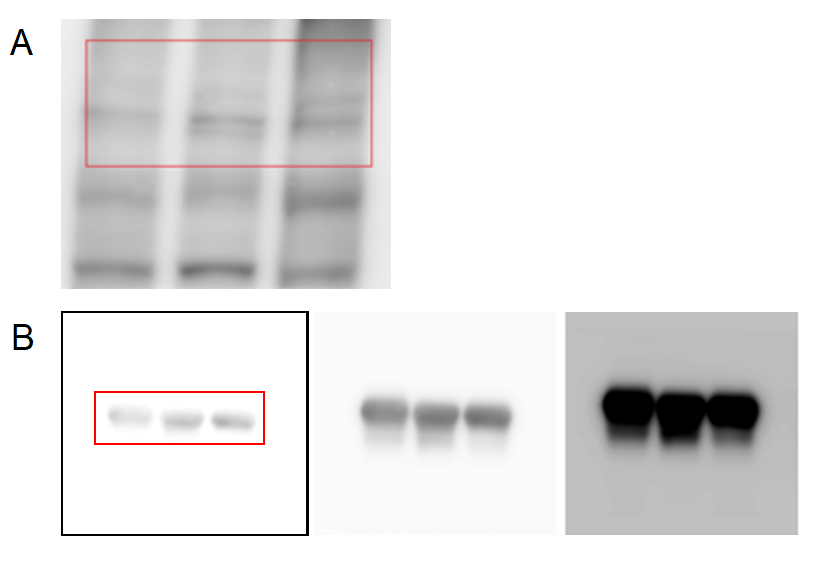


**Supplementary Figure 3**. Original and unprocessed blots of ABCA1western blot analysis (**A**) from Figure 4e and their internal control blots of α-tubulin (multiple exposure images, **B**). Showed blots at Figure 4e are marked with red boxes.


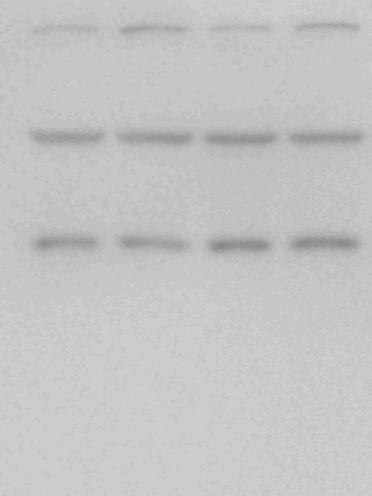


C


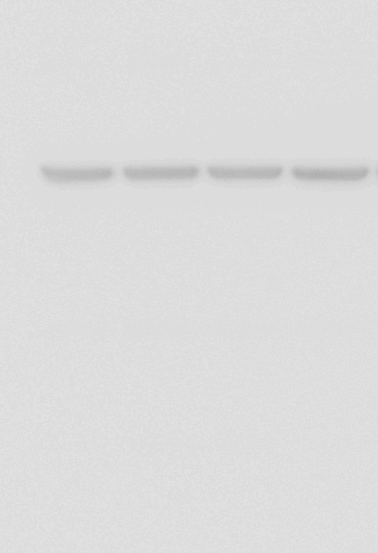


D


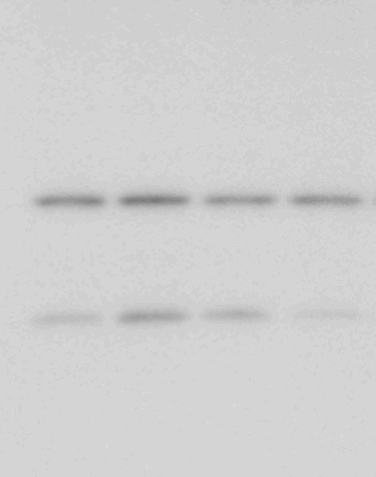


A


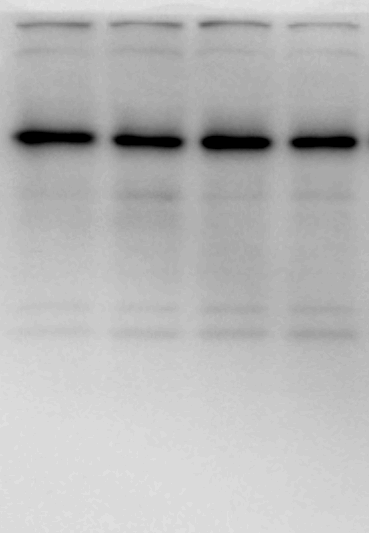


B

**Supplementary Figure 4**. Original and unprocessed blots of caspase-3 (p20) (**A**), caspase-9 (p39) (**B**), caspase-8 (p55) (**C**) and their internal control blots of α-tubulin (**D**) western blot analysis from Figure 5d. Showed blots at Figure 5d are marked with red boxes.
